# Adenosquamous carcinoma of the ovary arising from endometriosis: two case reports

**DOI:** 10.4076/1757-1626-2-6661

**Published:** 2009-08-04

**Authors:** Tadashi Terada

**Affiliations:** 1 Department of Pathology, Shizuoka City Shimizu Hospital, Shizuoka, Japan

## Abstract

The author reports two cases of adenosquamous carcinoma arising from endometriosis of ovaries. The tumor patients were 38-year-old and 53-year-old women. Both patients underwent hysterectomy and bilateral salpingo-oophorectomy for ovarian carcinomas. Grossly, both ovarian tumors were located in the left ovaries, and were cystic tumors with mural tumors. Histologically, the cystic areas consisted of endometrial glandular epithelium. Both mural tumors were composed of grade I endometroid adenocarcinoma and squamous cell carcinoma. These two elements were admixed in some areas. A differentiation of endometrioid adenocarcinoma from the endometriosis were present in a few areas. Likewise, a differentiation of squamous cell carcinoma from the endometriosis were recognized in several areas. The pathologic diagnosis was adenosquamous carcinoma arising from endometriosis of the ovary in both cases, rather than endometrioid adenocarcinoma with malignant squamous differentiation. No tumors were present in other organs.

## Introduction

It is well recognized that endometrosis may show malignant transformation [1]. Two of the most common malignant tumors of such a situation were clear cell adenocarcinoma and endometrioid adenocarcinoma [2]. The author herein reports two cases of adenosquamous carcinoma (ASC) of the ovaries. Since the squamous element of the two ovarian tumors was strongly suspected to be derived from endometrosis, the author used the term ASC rather than endometrioid adenocarcinoma with squamous differentiation.

## Case presentations

**Figure 1:**
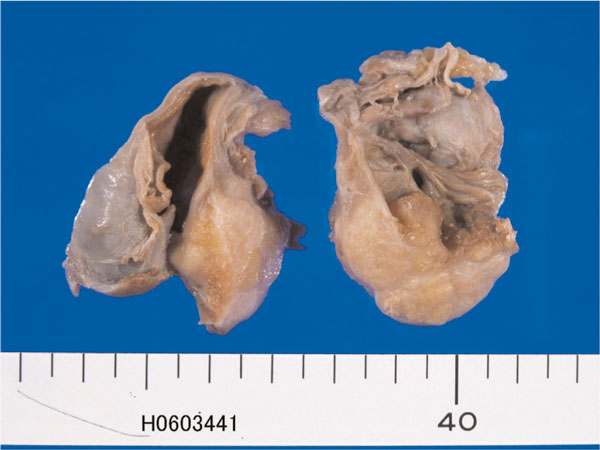
**Gross features of endometriosis of the ovary containing adenosquamous carcinoma. The tumor is cystic. The thicken wall area is a carcinoma, and thin wall area is endometriosis**.

**Figure 2:**
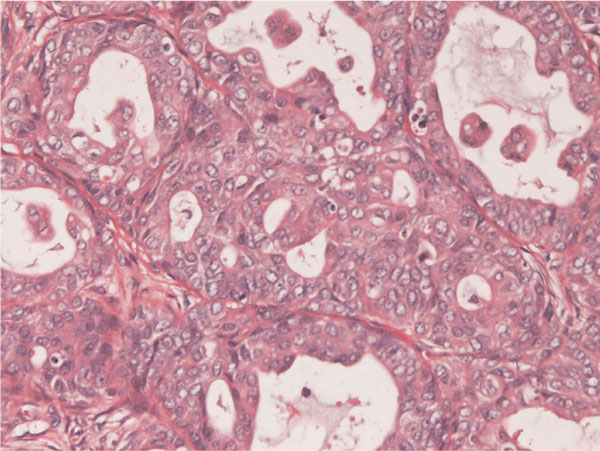
**Histopathology of adenosquamous carcinoma of the ovary arising from endometriosis**.

**Figure 3:**
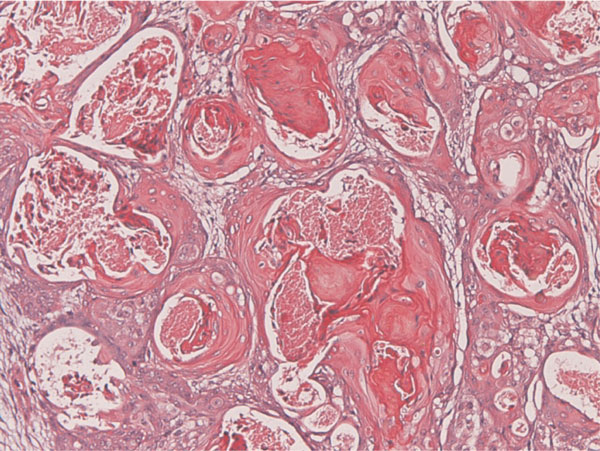
**Grade I endometrioid adenocarcinoma of grade I is present within the tumor**. HE, ×200.

**Figure 4:**
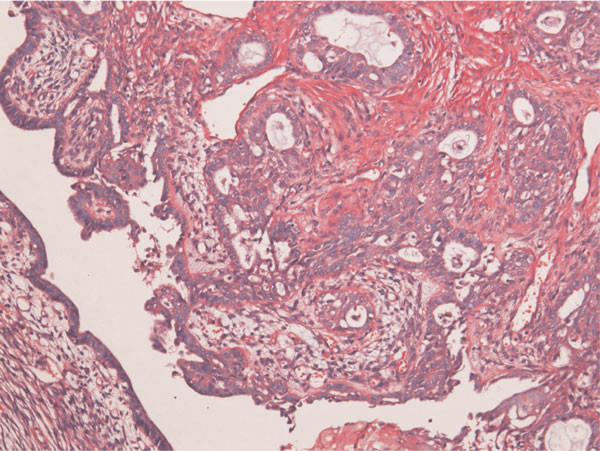
**Squamous cell carcinoma is recognized within the tumor**. C: A transition between endometorioid adenocarcinoma and endometriosis is present. This suggests that the endometrioid adenocarcinoma is derived from endometriosis. HE, ×200.

**Figure 5:**
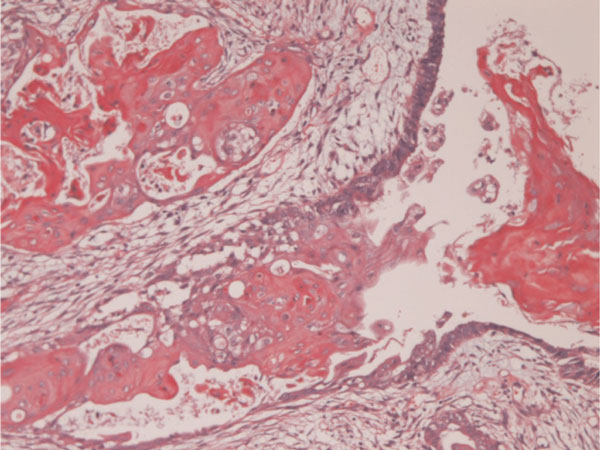
**A transition between squamous cell carcinoma and endometrosis is recognized**. This strongly suggests that the squamous cell carcinoma arise from endometriosis. HE, ×200.

The tumor patients were 38-year-old and 53-year-old Japanese women. Both patients underwent hysterectomy and bilateral salpingo-oohorectomy for ovarian carcinomas. Grossly, both ovarian tumors were located in the left ovaries, and were cystic tumors with mural tumors (Figure 1). Histologically, the cystic areas consisted of endometrial glandular epithelium. Both mural tumors were composed of grade I endometroid adenocarcinoma (Figure 2) and squamous cell carcinoma (Figure 3). These two elements were admixed in some areas. A differentiation of endometrioid adenocarcinoma from the endometriosis (Figure 4) were present in several areas. Likewise, a differentiation of squamous cell carcinoma from the endometriosis (Figure 5) was recognized in several areas. The pathologic diagnosis was ASC arising from endometriosis of the ovary in both cases, rather than endometrioid adenocarcinoma with malignant squamous differentiation. No tumors were present in other organs.

## Discussion

It is occasionally recognized that endometroid adenocarcinoma contains squamous element. Such tumors are now termed as endometrioid adenocarcinoma with squamous differentiation [3]. The squamous elements are morula, benign, borderline, and malignant. However, there is no clear evidence that the squamous cell carcinoma is a true squamous transformation. In the past, such cases were called ASC [4,5].

In the present two cases, transition between squamous cell carcinoma and endometriosis was recognized, strongly suggesting that squamous cell carcinoma element arose from endometriosis epithelium. Also, transition between adenocarcinoma element and endometriosis, suggesting that adenocarcinoma element is also derived from endometriosis epithelium. Therefore, both squamous cell carcinoma and adenocarcinoma elements are regarded to be derived from endometriosis epithelium; so the author used the term of ASC in both cases. The squamous cell carcinoma element and endometrioid adenocarcinoma element were admixed within the tumor. It may be also possible that squamous or adenocarcinomatous differentiation within the tumors.

In summary, the author presented two cases of ASC derived from endometriosis of the ovaries.

## Abbreviations

ASC: Adenosquamous carcinoma.

## Consent

Written informed consent was obtained from the patients for publication of this case report and accompanying images. A copy of the written consent is available for review by the Editor-in-Chief of this journal.

## Competing interests

The author declares that they have no competing interests.
